# The *fliK* Gene Is Required for the Resistance of *Bacillus thuringiensis* to Antimicrobial Peptides and Virulence in *Drosophila melanogaster*

**DOI:** 10.3389/fmicb.2020.611220

**Published:** 2020-12-18

**Authors:** Zaynoun Attieh, Carine Mouawad, Agnès Rejasse, Isabelle Jehanno, Stéphane Perchat, Ida K. Hegna, Ole A. Økstad, Mireille Kallassy Awad, Vincent Sanchis-Borja, Laure El Chamy

**Affiliations:** ^1^UR-EGP, Faculté des Sciences, Université Saint-Joseph de Beyrouth, Beirut, Lebanon; ^2^Université Paris-Saclay, INRAE, AgroParisTech, Micalis Institute, Jouy-en-Josas, France; ^3^Department of Pharmacy, Centre for Integrative Microbial Evolution (CIME), Faculty of Mathematics and Natural Sciences, University of Oslo, Oslo, Norway

**Keywords:** antimicrobial peptides, resistance, *Bacillus thuringiensis*, *Drosophila melanogaster*, virulence, FliK, flagella

## Abstract

Antimicrobial peptides (AMPs) are essential effectors of the host innate immune system and they represent promising molecules for the treatment of multidrug resistant microbes. A better understanding of microbial resistance to these defense peptides is thus prerequisite for the control of infectious diseases. Here, using a random mutagenesis approach, we identify the *fliK* gene, encoding an internal molecular ruler that controls flagella hook length, as an essential element for *Bacillus thuringiensis* resistance to AMPs in *Drosophila*. Unlike its parental strain, that is highly virulent to both wild-type and AMPs deficient mutant flies, the *fliK* deletion mutant is only lethal to the latter’s. In agreement with its conserved function, the *fliK* mutant is non-flagellated and exhibits highly compromised motility. However, comparative analysis of the *fliK* mutant phenotype to that of a *fla* mutant, in which the genes encoding flagella proteins are interrupted, indicate that *B. thuringiensis* FliK-dependent resistance to AMPs is independent of flagella assembly. As a whole, our results identify FliK as an essential determinant for *B. thuringiensis* virulence in *Drosophila* and provide new insights on the mechanisms underlying bacteria resistance to AMPs.

## Introduction

The continuous emergence of new infectious agents, together with the worrying rise in antibiotic resistance in pathogens, constitutes a threat to human health with predictions that these will account for 20% of deaths over the coming decades (ECDC/EMEA, 2009). Indeed, in the last 60 year*s*, no fewer than 335 new infectious diseases of humans have emerged (Jones et al., 2008) and 30% of all emerging infections were comprised of pathogens transmitted through food (Kuchenmuller et al., 2009). Seventy percent of germs responsible for infections in hospitals are resistant to at least one class of antibiotics and the emergence of strains resistant to several classes of antibiotics is beginning to pose serious therapeutic problems (Levy and Marshall, 2004). These data highlight the urgent need to implement new therapeutic strategies and develop new classes of antimicrobial agents to control infections. In this perspective, the discovery in the 90s of antimicrobial peptides (AMPs) in animals as part of their innate immune defenses has paved the way for a new and very broad field of research. AMPs are small, cationic, usually amphipathic peptides with a broad spectrum of activity against Gram-positive and Gram-negative bacteria, fungi, viruses, and protozoa (Imler and Bulet, 2005; Guani-Guerra et al., 2010; Marxer et al., 2016; Rolff and Schmid-Hempel, 2016; Haney et al., 2017; Hanson and Lemaitre, 2020). *Ex-vivo* studies have shown that their potent antimicrobial activity is exhibited mostly by perturbing negatively charged microbial membranes, although some AMPs can also act at intracellular targets preventing the synthesis of essential elements for biological functioning and the survival of microorganisms (Park et al., 1998; Kragol et al., 2001; Brogden, 2005; Rahnamaeian et al., 2015; Scocchi et al., 2016; Yang et al., 2019). Therefore, AMPs constitute a promising alternative therapeutic option that should make it possible to provide new classes of molecules with a broad spectrum of antimicrobial activity to possibly minimize or delay the onset of resistance (Andres and Dimarcq, 2004; Zaiou, 2007). However, if AMPs are taken out of their natural environment and used as antimicrobial agents in a conventional clinical setting, they could exert a strong and continuous selection pressure on the bacterial population leading to rapid selection of resistant strains. Such a scenario could have extremely serious consequences if it resulted in the development of cross-resistance with innate human AMPs (Bell and Gouyon, 2003; Perron et al., 2006). Therefore, an accurate knowledge of the mechanisms of resistance to these compounds, and a better understanding of the environmental constraints encountered by pathogenic bacteria during the infectious process, are prerequisites to their intensive use.

The *Bacillus cereus* group includes a growing number of species which range from no hazardous to deadly dangerous (Carroll et al., 2020). To date 21 genomospecies have been described (Liu et al., 2017). These include *B. anthracis*, the infamous causing agent of the anthrax disease, the entomopathogen *B. thuringiensis* that is widely used as a biological control agent to combat insect pests of agriculture or vectors of diseases and *B. cereus sensu stricto* an opportunistic human pathogen which causes gastroenteritis and is now considered as the third most important cause of collective food poisoning incidents in Europe (EFSA, 2009). These Gram-positive bacteria are ubiquitous in nature and their spores are highly resistant to common sterilizing techniques which make them of a high concern to the food industry. The ability of *B. cereus* group members to form biofilms adds complexity and persistence to bacteria on industrial and biomedical devices which are currently recognized as defining sources for nosocomial infections (Stenfors Arnesen et al., 2007; Kuroki et al., 2009; Bottone, 2010; Glasset et al., 2018). Although *B. cereus* food-borne poisoning is generally mild, it is capable of causing serious gastrointestinal diseases in humans, such as bloody diarrhea and emetic poisoning, eventually leading to some fatal cases (Mahler et al., 1997; Lund et al., 2000; Dierick et al., 2005), especially in immunocompromised patients and preterm neonates (Stenfors Arnesen et al., 2008). Most alarming is the fact that *B. cereus* is now also increasingly diagnosed as a cause of severe, frequently fatal, non-gastrointestinal infections such as: bacteremia (Hernaiz et al., 2003), osteomyelitis (Sliman et al., 1987), septicemia (Matsumoto et al., 2000), pneumonia (Gray et al., 1996; Miller et al., 1997), liver abscess (Latsios et al., 2003). More recently, a novel food pathogen, *B. cytotoxicus* (Lund et al., 2000; Fagerlund et al., 2007; Auger et al., 2008; Lapidus et al., 2008; Guinebretiere et al., 2013), as well as *B. cereus* “*anthracis*-like” strains were also characterized (Hoffmaster et al., 2004; Klee et al., 2010), showing emergence of new pathotypes in the group. Although distinctive pathogenic features of the *B. cereus* group species are linked to plasmid-born genes specifying their susceptible hosts, these bacteria share a common genetic background with several genes associated with the expression of their virulence phenotypes (Ehling-Schulz et al., 2019). Indeed, the opportunistic properties of *B. cereus* and *B. thuringiensis* have been explored in animal models underlying the requirement of common genetic determinant in the pathogenic properties of these bacteria (Salamitou et al., 2000; Gohar et al., 2008; Ramarao et al., 2012; Ramarao and Sanchis, 2013). In agreement with this hypothesis, some *B. thuringiensis* strains have been reported to cause infections in immunocompromised patients (Green et al., 1990; Damgaard et al., 1997; Hernandez et al., 1998; Helgason et al., 2000; Kuroki et al., 2009). These data emphasize the necessity of an in-depth investigation of the common genetic determinants involved in the determination of the pathogenic potential of *B. cereus* species.

Previous studies have reported the prominent feature of *B. cereus* and *B. thuringiensis* to rapidly develop in insect models upon septic injury infection (Stephens, 1952; Kushner and Heimpel, 1957; Salamitou et al., 2000; Buisson et al., 2019). These data presumably underlined the potent capacity of these bacteria to overcome host innate immune defenses. In agreement with these findings, we have previously reported that *B. cereus* is highly resistant to AMPs (Abi Khattar et al., 2009). This resistance relies on the D-alanyl esterification of its cell wall teichoic acids (TAs) through the activity of the genes products of the *dltXABCD* operon that is highly conserved among Gram-positive bacteria (Heaton and Neuhaus, 1992; Perego et al., 1995; Peschel et al., 1999; Abachin et al., 2002; Kristian et al., 2005; Abi Khattar et al., 2009; Kamar et al., 2017). The D-alanylation of TAs reduces their net negative charge, thus lowering the attraction of cationic AMPs to the bacterial cell wall (Kristian et al., 2003, 2005; Fabretti et al., 2006; Kovacs et al., 2006; Perea Velez et al., 2007). We have further explored the relevance of *B. cereus* resistance to AMPs in the context of its exposure to the host immune defenses using the *Drosophila* model. Indeed, in insects as in mammals, sensing of Microbial Associated Molecular Patterns (MAMPs) by cognate host innate immune Pattern Recognition Receptors (PRRs) triggers a highly conserved immune response that is prerequisite to counter the infections (Janeway, 1989; Medzhitov, 2007). In *Drosophila*, sensing of *Bacilli* Diaminopimelic acid (DAP)-type peptidoglycan triggers the activation of the immune deficiency (IMD) pathway which controls the activation of the NF-κB transcription factor, Relish, thus driving the expression of several immune genes including those encoding AMPs (Leulier et al., 2003; Kaneko et al., 2004; Stenbak et al., 2004). Seven AMP families have been characterized in *Drosophila* (Imler and Bulet, 2005; Hanson and Lemaitre, 2020). Their highly induced expression upon microbial challenge, provides microbicidal concentrations that are far above the concentration necessary to kill bacteria (Hanson et al., 2019; Hanson and Lemaitre, 2020). Using a septic injury infection model, we have shown that *B. thuringiensis* is highly resistant to the *Drosophila* Relish-dependent systemic antimicrobial response. Remarkably, contrary to the wild-type (wt) bacterial strain, that is equally virulent to both wt and *relish* mutant flies, a *B. thuringiensis Δdlt* mutant only exhibited high pathogenicity in *relish* immunodeficient flies (Kamar et al., 2017).

Here, taking advantage of this *Drosophila* model of bacteremia which easily allows the discrimination between virulent and attenuated bacterial strains, together with the recent development of an AMP-deficient *Drosophila* strain (Hanson et al., 2019), we aimed at further exploring physiological and genetic factors that make *B. cereus* resistant to host AMPs *in vivo*. Using a random mutagenesis approach, we identified the *fliK* gene, which encodes a protein with a flagellar hook length control motif, as an essential determinant for *B. thuringiensis* resistance to AMPs and virulence in *Drosophila*. In agreement with its highly conserved function, the *fliK* mutant is non-flagellated and exhibits highly compromised motility. However, by combining both *in vitro* and *in vivo* analysis, we show that *B. thuringiensis* FliK-dependent resistance to AMPs is independent of the flagellar motility. As a whole, our results, provide new insights on the strategies developed by bacteria to overcome host innate immune defenses, in particular AMPs, which could be exploited for potential therapeutic approaches to decrease viability of pathogens.

## Results

### A Two-Step Screening Strategy Identifies Genes Required for the Resistance of *Bt407* to Polymyxin B and to Innate Immune Defenses in *Drosophila*

In an attempt to identify new genes involved in the resistance of *B. cereus* to innate immune defenses, and more specifically to AMPs, we generated, by insertion and mobilization of a mini-Tn*10* transposon, a random mutagenesis library of the *B. thuringiensis Bt407* strain (Lereclus et al., 1989) that we screened in a two-step strategy as described in the following. In the first step of the screen, *Bt407^*mini*–*Tn10*^* insertion mutants were screened on LB-Agar plates supplemented with 200 μg/ml of polymyxin B in order to select clones with a growth delay comparable to that of the *dlt* operon mutant, *Bt407*Δ*dltX* (Kamar et al., 2017). Out of 3,200 tested clones, this primary screen allowed the selection of 10 *Bt407^*mini*–*Tn10*^* insertion mutants with a significant growth delay on polymyxin B as compared to the wt strain. The phenotype of each of the selected clones was confirmed by three independent experiments. To exclude the possibility that the growth delay phenotype observed in the selected *Bt407^*mini*–*Tn10*^* insertion mutants was due to a growth deficiency rather than an increased sensitivity to polymyxin B, their growth was assessed in LB broth medium at 30°C. All mutants grew in the same way as the *Bt407* strain in the absence of polymyxin B. In contrast, their growth was affected during exponential phase when the culture medium was supplemented by polymyxin B at 200 μg/ml. Mapping of the transposon insertion sites (see section “Materials and Methods”) revealed that, in all mutants, the transposon was inserted in an open reading frame (ORF) flanked by 9 bp duplication at each end, which is a mini-Tn*10* insertion characteristic. The 10 insertions mapped to four distinct ORFs that were identified and designated PSC1 to PSC4 (for Polymyxin B Sensitive Clone). The insertions in the identified ORFs hit the following genes, respectively, *BTB_c16930* encoding a hypothetical protein with a flagellar hook length control protein motif, the *BTB_c20220* gene encoding the cell wall-associated hydrolase LytF1, the gene *BTB_c28480* encoding the NprM bacillolysin and the *BTB_c32720* gene encoding a hypothetical protein with a ß-lactamase motif characteristic of penicillin-binding proteins (Table 1).

**TABLE 1:** Loci of mini-Tn*10* insertions in the selected polymyxin B sensitive clones.

**Mutant clone**	**Mini-Tn*10* interruption gene**	**Annotation**
*PSC1*	*BTB_c16930*	Hypothetical protein (with flagellar hook length control protein motif)
*PSC2*	*BTB_c20220*	Endopeptidase, a cell wall associated hydroslase (LytF1)
*PSC3*	*BTB_c28480*	Bacillolysin, a *Bacillus* metalloendopetitase (NprM)
*PSC4*	*BTB_c32720*	Hypothetical protein (with a ß-lactamase motif characteristic of penicillin-binding proteins)

**FIGURE 1:**
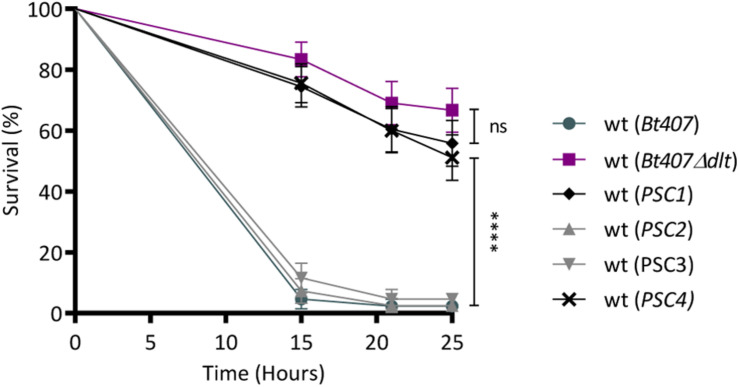
Identification of novel genes involved in *Bt407* virulence in *Drosophila*. Survival of wild-type flies (wt) to an infection with *Bt407*, *Bt407*Δ*dlt* or the four *Bt* mutants preselected as Polymyxin B Sensitive Clones (*PSC*). Data are representative of three independent experiments (mean ± SD). Statistical tests were performed using Log Rank test (ns: *p* > 0.05, ^****^p < 0.0001).

To address the primary objective of this study, which is the identification of genes involved in the resistance to innate immune defenses, in the second step of the screen, we evaluated the virulence of the four *PSC* insertion mutants upon a septic injury infection in adult *Drosophila*. This infection model, previously established on the wt and the *dlt* mutant strains of *Bt407*, allows the discrimination between virulent and attenuated strains (Kamar et al., 2017; Figure 1). Thus, we compared the survivals of wt flies infected with the *PSC* clones to those of flies infected with the wt and the *dlt* mutant strains of *Bt407*. The results indicate that, similarly to the *Bt407*Δ*dlt* mutant, the *PSC1* and *PSC4* clones had an attenuated virulence when compared to the parental *Bt407* strain (Figure 1). Overall, these results allowed the selection of two candidate genes required for the resistance of *Bt407* to polymyxin B in culture medium and to host defenses in *Drosophila*. Based on gene mapping and homology sequence analysis, the candidate genes interrupted by the mini-Tn*10* transposon in the *PSC1* and *PSC4* clones are likely involved in flagellar and cell wall assembly, respectively. Several studies have associated the flagellum to different aspects of bacterial virulence (Duan et al., 2013). In *Campylobacter jejuni*, locomotion and resistance to AMPs have also been correlated (Cullen and Trent, 2010; Cullen et al., 2012). These traits were separately attributed to the dual function of a phosphoethanolamine transferase (EptC) which modifies both the flagellar rod protein FlgG and the lipid A domain of lipooligosaccharide, respectively (Cullen et al., 2012). However, a direct link between flagella and the resistance of bacteria to AMPs remains largely unexplored. Therefore, in this study we aimed at investigating the role of flagella in the resistance to AMPs with a particular focus on the role of the *BTB_c16930* gene.

### The *fliK* Gene Is Required for *Bt407* Flagellar Filament Assembly and Function

The bacterial flagellum is a complex organelle that projects from the inside-out of the cell. It comprises three functional parts: (1) the basal body, which is embedded in the cell envelope, and that comprises a stator, a rotor, and an axle-like rod that extends through the cell wall peptidoglycan and houses a type III secretion system that exports the more distal components of the organelle, (2) the hook, which constitutes a flexible universal joint connecting the basal body to the last part of the flagellum, and (3) the helical filament which acts as a propeller (Guttenplan and Kearns, 2013; Evans et al., 2014; Mukherjee and Kearns, 2014). According to the KEGG database (Kyoto Encyclopedia of Genes and Genomes), the *BTB_c16930* gene encoding a hypothetical protein is the ortholog of *fliK* in *B. thuringiensis HD1011* strain with 91.1% of protein sequence identity. Functional analysis performed in *Bacillus subtilis* and Gram-negative bacteria have shown that *fliK* encodes an internal molecular ruler which controls hook length (Silverman and Simon, 1972; Patterson-Delafield et al., 1973; Minamino et al., 1999b, 2004; Journet et al., 2003; Shibata et al., 2007; Chevance and Hughes, 2008; Erhardt et al., 2010; Courtney et al., 2012). Furthermore, as the secretion of flagellar components is sequential, FliK was also shown to be required for switching the specificity of the export machinery from rod-hook substrates to flagellin, the protein products of the *fla* genes in *Bt407*, thereby promoting extracellular filament assembly (Moriya et al., 2006; Minamino et al., 2009; Houry et al., 2010; Erhardt et al., 2011; Mizuno et al., 2011; Hughes, 2012a,b; Evans et al., 2013). This is achieved once the hook has reached its mature length.

**FIGURE 2:**
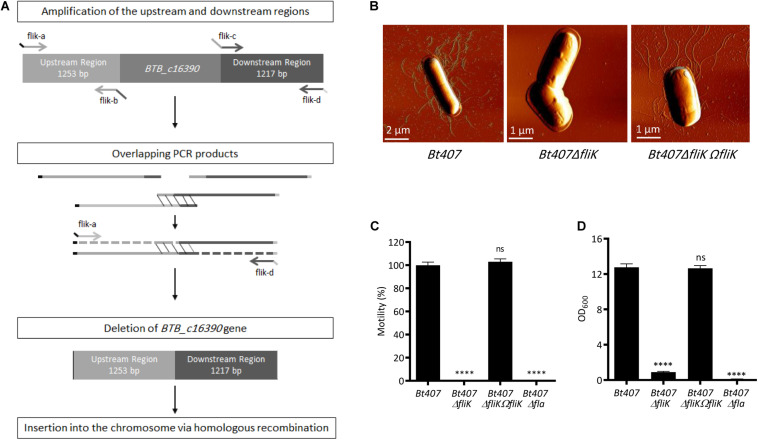
FliK is required for *Bt407* flagellar assembly. **(A)** Schematic diagram showing the construction of an in-frame deletion of the *BTB_c16930* gene by Splicing by Overlap Extension (SOE). **(B)** Scanning Atomic Force Microscopy (AFM) of exponential growth phase cells of *Bt407*, *Bt407*Δ*fliK*, and *Bt407*Δ*fliKΩfliK*. **(C)** Bacterial motility on LB soft agar (0.25%) medium. Motility of the *Bt407* strain was set to 100%, and values obtained with other strains were set as fold relative to this value. **(D)** Total biofilm biomass at the air-liquid interface in glass tubes, following 48 h incubation at 30°C. Data obtained from three independent experiments are combined in single value (mean ± SD). Statistical tests were performed using the Mann-Whitney test within Prism software (ns: *p* > 0.05; ^****^p < 0.0001).

In order to confirm the role of the *fliK* gene in the assembly of flagella in *Bt407*, and to avoid any possible effect arising from the mini-Tn*10* mobilization or unrelated secondary mutation, we constructed a *de novo Bt407ΔfliK* deletion mutant harboring a precise deletion of the *BTB_c16930* gene (Figure 2A) and its complemented strain *Bt407*Δ*fliKΩfliK*. Using scanning atomic force microscopy (AFM), we examined the presence of flagella on *Bt407*Δ*fliK*. As shown in Figure 2B, unlike the wt *Bt407* strain, the *Bt407*Δ*fliK* deletion mutant completely failed to produce detectable flagellar filaments as shown in amplitude images (Figure 2B). No loose fragments of flagella were observed in the mutant cellular preparation as shown in amplitude images. In agreement with these findings, we showed that, similarly to the *Bt407*Δ*fla* mutant (Houry et al., 2010), in which the genes encoding flagellar proteins had been interrupted, the *Bt407*Δ*fliK* mutant shows a highly compromised swimming motility as compared to the wt *Bt407* strain (Figure 2C). Both flagellar assembly and swimming motility are restored by the functional complementation of the *Bt407*Δ*fliK* deletion mutant by the *fliK* ORF (Figures 2B,C). Altogether, these results confirm the essential function of the predicted FliK protein in the flagellar assembly and associated motility of *Bt407*. Since flagella mediated motility was shown to promote biofilm formation for several bacterial species including *B. thuringiensis* (O’Toole and Kolter, 1998; Pratt and Kolter, 1998; Klausen et al., 2003; Wood et al., 2006; Lemon et al., 2008; Houry et al., 2010, 2012; Guttenplan and Kearns, 2013; Fagerlund et al., 2014; Majed et al., 2016) and since biofilms are known to enhance bacterial resistance to antimicrobial effectors (Costerton, 2004; Hall-Stoodley and Stoodley, 2009), we sought to evaluate the capacity of the *Bt407*Δ*fliK* mutant to form a biofilm at the air-liquid interface in a glass tube. Quantification of biofilm biomasses presented in Figure 2D clearly indicates that, similarly to the *Bt407*Δ*fla* mutant, biofilm formation is highly compromised for the *Bt407*Δ*fliK* mutant. This phenotype was reverted in the complemented *Bt407*Δ*fliKΩfliK* strain (Figure 2D). This result underlies the requirement of the FliK protein for flagellar associated biofilm formation in *Bt407*.

### Beyond Its Role in Flagellar Assembly, FliK Is Essential for the Resistance of *Bacillus thuringiensis* to Host Anti Microbial Peptides

**FIGURE 3:**
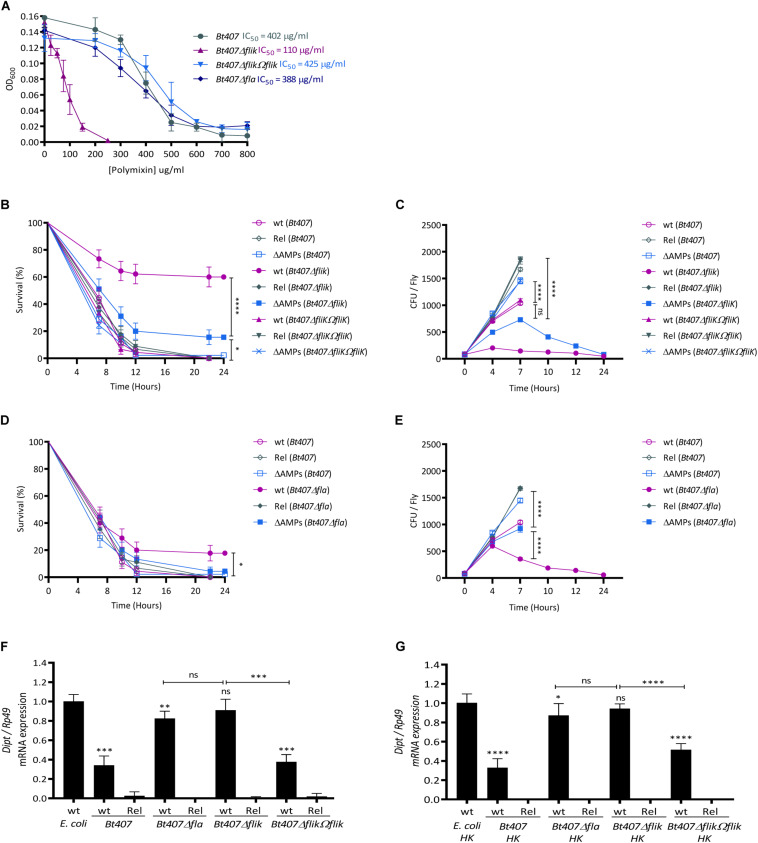
FliK confers *Bt407* resistance to *Drosophila* antimicrobial peptides in a flagella independent manner. **(A)** The half inhibitory concentration (IC50) of *Bt407*, *Bt407*Δ*fliK*, *Bt407*Δ*fliKΩfliK*, and *Bt407*Δ*fla*. The bacterial growth was scored 6 h post incubation at 30°C in LB medium supplemented with increased concentrations of polymyxin B. **(B,D)** Survival of adult wild-type (wt), *relish* (*Rel*) and antimicrobial peptides (Δ*AMPs*) mutant flies to an infection with *Bt407* **(B,D)**, *Bt407*Δ*fliK* **(B)**, *Bt407*Δ*fliKΩfliK* **(B)** or *Bt407*Δ*fla* **(D)**. **(C,E)** Internal bacterial loads retrieved from adult flies infected with *Bt407* **(C,E)** *Bt407*Δ*fliK* **(C)**, *Bt407*Δ*fliKΩfliK* **(C),** or *Bt407*Δ*fla* **(E)**. The Colony Forming Unit (CFU) counting was performed only on surviving flies. **(F,G)** Relative expression of the *Diptericin* (*Dipt*) transcripts in wild-type (wt) or *relish* (*Rel*) mutant flies induced by living **(F)** or heat-killed HK **(G)** *Bt407, Bt407ΔfliK*, *Bt407*Δ*fliKΩfliK*, or *Bt407*Δ*fla.* Transcripts expression was measured by RT-qPCR in total RNA extracts 4 h upon the induction. Ribosomal protein 49 (Rp49) transcript was used as reference gene. Transcripts levels are compared to that triggered in wt flies infected by living **(F)** or heat-killed HK **(G)** *E. coli* as a control. Data obtained from three independent experiments are combined in single value (mean ± SD). Statistical tests were performed using the Log Rank test for the survival assays and Mann-Whitney test for the CFU counting and the *Dipt* expression evaluation within Prism software (ns: *p* > 0.05; ^∗^0.01 < *p* < 0.05; ^∗∗^0.001 < *p* < 0.01; ^∗∗∗^0.0001 < *p* < 0.001; ^****^p < 0.0001).

In order to confirm that the enhanced sensitivity of the *PSC1* clone to polymyxin B is due to the compromised function of the *fliK* gene, we assessed the susceptibility of the *Bt407*, *Bt407*Δ*fliK*, and *Bt407*Δ*fliKΩfliK* strains to polymyxin B by determining their half inhibitory concentration (IC50). As presented in Figure 3A, the *Bt407*Δ*fliK* mutant is highly sensitive to polymyxin B with an IC50 fourfold lower than that of *Bt407* and *Bt407*Δ*fliKΩfliK* strains (IC50s of 110, 402, and 425 μg/ml, respectively) (Figure 3A). To check whether the increased sensitivity to polymyxin B observed in *Bt407*Δ*fliK* was due to the mutation of the *fliK* gene or more generally to a flagellar filament production deficiency, we henceforth included in our experiment the *Bt407*Δ*fla* mutant (Houry et al., 2010). The results indicate that, the growth of the *Bt407*Δ*fla* mutant is comparable to that of the parental *Bt407* strain with an IC50 of 385 μg/ml for polymyxin B *in vitro* (Figure 3A).

We then sought to check whether the attenuated virulence of the *Bt407*Δ*fliK* was also due to the FliK-dependent resistance of *Bt407* to host AMPs. Therefore, we assessed the survival of wt and Δ*AMPs* mutant flies, which are devoid of 10 out of the 14 known *Drosophila* immune inducible AMPs encoding genes, to an infection by *Bt407*, *Bt407*Δ*fliK, Bt407ΔfliKΩfliK*, or *Bt407*Δ*fla* (Hanson et al., 2019). We also included in the analysis *relish* mutants which are unable to mount an IMD-dependent systemic humoral response upon the infection of flies by *Bacillus* spp. (Leulier et al., 2003). As shown in Figure 3B, unlike the wt *Bt407* strain, which is highly resistant to the Relish-dependent AMPs response in flies thus killing wt, Δ*AMPs* and *relish* mutants with the same kinetics (approximately 100% of lethality 12 h post-infection), the *fliK* mutant displays an attenuated virulence in immunocompetent flies (37% of lethality at 12 h post-infection) while remaining fully virulent in immunodeficient flies sharing the same lethality kinetics with its parental strain (Figure 3B). The survival of Δ*AMPs* mutant flies largely mirrored that of *relish* mutants thus attesting of the sensitivity of this mutant to AMPs effectors among all Relish-immune induced genes. The slight difference between the survival of *relish* and Δ*AMPs* mutants could be explained by the expression in the latter of the four remaining genes encoding antibacterial Cecropins which are known to be induced by the IMD pathway (Leulier et al., 2000). The sensitivity of the *Bt407*Δ*fliK* mutant to AMPs is further attested by the quantification of bacterial loads in the hemolymph of Δ*AMPs* mutants as compared to wt flies (Figure 3C). Indeed, whereas *Bt407*Δ*fliK* is unable to grow in wt flies, its growth in Δ*AMPs* mutants is equivalent to that of the parental *Bt407* strain in wt flies up to 7 h post infection (Figure 3C). Passing this time point, the growth of *Bt407*Δ*fliK* in the hemolymph of Δ*AMPs* flies is likely compromised by the accumulated expression of Cecropins. This hypothesis is further supported by its accentuated growth in the hemolymph of *relish* mutants which are devoid of all IMD-dependent AMPs, and the concomitant lethality of these flies to the infection. These phenotypes are strictly dependent on the activity of FliK as revealed by the virulence phenotype and the growth of the *Bt407*Δ*fliKΩfliK* complemented strain in both wt and Δ*AMPs* mutant (Figures 3B,C). Interestingly, in agreement with its resistance to polymyxin B phenotype, the *Bt407*Δ*fla* mutant showed a virulence phenotype that contrasts with that of the *Bt407*Δ*fliK*. In particular, the *Bt407*Δ*fla* mutant remained highly virulent with wt flies dying most similarly to Δ*AMPs* and *relish* mutants up 12 h post infection (Figure 3D). Comparative analysis of the growth of bacterial strains in the hemolymph of these flies, shows that unlike *Bt407*Δ*fliK*, *Bt407*Δ*fla* grows similarly to the wt strain up to 4 h after the infection (Figure 3E). This result correlates with an identical lethality rate of the flies 7 h after the infection. Passing this time point, a slightly attenuated virulence is observed with only 20% of wt flies surviving 12 h post-infection (Figure 3D). This decreased virulence of the *Bt407*Δ*fla* is correlated with a decrease of its growth in the hemolymph of wt flies as compared to the parental strain (Figures 3D,E). Thus, compared to the *Bt407*Δ*fliK* mutant that is completely compromised by the *relish*-dependent immune response, a *Bt407*Δ*fla* mutant is only sensitive to this response at later time points of the infection when the bacterial virulence phenotype is already prominently expressed (compare bacterial growth in wt and *relish* mutant flies). These data suggest that the additive expression of Relish-dependent AMPs encoding genes might account for the slight, although significant reduced virulence of *Bt407*Δ*fla* in wt as compared to *relish* mutants’ flies. Interestingly, the early growth of *Bt407*Δ*fla* is similar to that of the parental *Bt407* strain in the hemolymph of wt flies. Thus, it is tempting to speculate that the continuing growth of the wt *Bt407* strain, passing 4 h of the infection, would account for its accentuated virulence on wt flies as compared to that of *Bt407*Δ*fla* (Figures 3D,E). Interestingly, the growth of the parental *Bt407* strain also seems to be slowed down by the Relish-dependent immune response 7 h after the infection (Figures 3C,E). However, the resistance of *Bt407* to this response is manifested by its continuous albeit attenuated growth but also by its fully virulent phenotype on wt, Δ*AMPs* and *relish* mutant flies (Figures 3B–E). Although these results provide clear evidence of a role of FliK in the resistance of *B. thuringiensis* to the *Drosophila* systemic AMPs Relish-dependent humoral response, this hypothesis is challenged by the possibility that the wt and mutant bacteria strains might induce a different immune response in the infected flies. In order to check for this possibility, we compared the immune response induced by the different bacterial strains, by quantifying the expression of *Diptericin*, an AMP-encoding gene, which is conventionally used as a readout of the Relish-dependent immune response in *Drosophila*. As shown in Figure 3F, *Bt407* induces a mild-immune response in *Drosophila* compared to the Gram-negative bacterium *Escherichia coli* that is commonly used as an IMD pathway inducer (Figure 3F). Both *Bt407*Δ*fla* and *Bt407*Δ*fliK* mutants trigger an enhanced immune response as compared to the parental or *Bt407*Δ*fliKΩfliK* strains. The reduced immune response triggered by *Bt407* is not due to an inhibitory mechanism depending on the activity of the flagella as the immune induced response profile remained unchanged whether the flies were treated with living or heat-killed bacteria (Figures 3F,G). Thus, these results rule out the possibility that the different virulence phenotypes of *Bt407*Δ*fliK* (37% of lethality) and *Bt407*Δ*fla* (80% of lethality) are due to a more prominent induction of immune effector genes in the host by the former. However, these data could potentially explain the slightly reduced virulence of *Bt407*Δ*fla* (20% of survivors) compared to that of the wt *Bt407* strain (0% survivors) at later timepoints of the infection where the higher and cumulative production of immune effector molecules might exert an additive effect on the bacteria thus limiting their growth in the hemolymph of the infected flies. Altogether, our results so far confirm a role of FliK in the resistance of *B. thuringiensis* to polymyxin B *in vitro* and to the *Drosophila* AMPs *in vivo* independently of its function in the establishment of flagella.

## Discussion

AMPs are produced by virtually all living organisms and their widespread role in innate immune defense has been largely recognized. They allow unicellular organisms to successfully compete with other organisms sharing their habitats and constitute key effectors of the innate immune system in metazoans (Schmitt et al., 2016; Ageitos et al., 2017). By acting at the frontline of host defenses, these microbicidal molecules play an essential role in limiting the infections. The development of resistance mechanisms to this immune arsenal is thus considered as a major virulence phenotype providing successful human pathogens the potential to produce serious invasive infections (Koprivnjak and Peschel, 2011; Cole and Nizet, 2016). Several studies have shown that bacterial pathogens have evolved different resistance mechanisms to AMPs, mainly by surface charge modification (Peschel et al., 2001; Thedieck et al., 2006; Samant et al., 2009; Nawrocki et al., 2014; Kamar et al., 2017). Here, we have combined *in vitro* and *in vivo* approaches for the identification of novel genes required for the resistance of *B. thuringiensis* to cationic AMPs. Screening of 3,200 clones of a random mini-T*10* insertional mutagenesis library allowed the selection of four clones with a compromised resistance to polymyxin B *in vitro*. Sequence analysis of the insertion sites of the transposon showed that these proteins were mainly secreted, or cell wall proteins associated with functional aspects of the cell wall and cell envelope. Of these mutants, only two exhibited a reduced virulence compared to the *Bt407* parental strain in a systemic infection model in *Drosophila*. These results emphasize the existence of various AMP resistance mechanisms in *B. thuringiensis*. These are likely to support the ubiquitous nature of this bacterium that is highly spread in the environment. These results also advance the importance of using *in vivo* infection models for the evaluation of the significance to pathogenesis of genetic variants that impact bacterial resistance to AMPs while confronted to the complex immune defenses in eukaryotic hosts (Bauer and Shafer, 2015).

In this study, we focused on one mutant that has a transposon insertion in the gene annotated as *fliK.* Phenotypic characterization of a precise *Bt407*Δ*fliK* deletion mutant allowed us to confirm that the *fliK* gene is a defining element of *B. thuringiensis* virulence in *Drosophila* with a pleiotropic phenotype. Indeed, unlike its parental strain, the *Bt407*Δ*fliK* mutant is non-flagellated and exhibits compromised motility and biofilm formation. These data are in agreement with the conserved essential function of *fliK* in flagellar assembly that was previously described for several bacterial species (Silverman and Simon, 1972; Patterson-Delafield et al., 1973; Journet et al., 2003; Minamino et al., 2004; Shibata et al., 2007; Chevance and Hughes, 2008; Erhardt et al., 2010; Courtney et al., 2012). The novelty of this report relies on the demonstration that the reduced pathogenicity of the *Bt407*Δ*fliK* mutant is largely due to its high sensitivity to AMPs independently of its role in flagellar assembly. Indeed, comparative analysis reveals striking difference between the phenotypes of *Bt407*Δ*fliK* and *Bt407*Δ*fla* mutants in terms of virulence in the *Drosophila* systemic infection model while both mutants share similar traits in terms of absence of flagella, reduced motility and biofilm formation. The highly attenuated virulence of *Bt407*Δ*fliK* compared to that of *Bt407*Δ*fla* is nonetheless associated with a marked enhanced susceptibility of the former (and not of the latter) to AMPs both *in vitro* and *in vivo*. In particular, our data show that Flik is essential for the resistance of wt *Bt407* strain to AMPs thus providing it with the advantage of a promoted growth from the early time of the infection. Indeed, timescale analysis indicates that the induced AMP humoral response efficiently inhibits the growth of *Bt407*Δ*fliK* in the hemolymph of infected flies but is unable to contain the early growth of the *Bt407*Δ*fla* mutant which remained comparable to that of the wt *Bt407* strain. Although the induced AMP response partially contains the growth of *Bt407*Δ*fla* in the hemolymph of wt flies during the progress of the infection, the early bacterial growth is sufficient to induce significant lethality of the flies in a manner very similar to those infected by the wt *Bt407* strain (Figures 3D,E). These results highlight the essential role of the FliK-dependent resistance to host AMPs in the virulence of *Bt407* in the *Drosophila* model, independently of its role in flagellar assembly and motility. Nevertheless, unsuccessful flagellar assembly seems to influence the sensing of bacteria by the host immune system. Indeed, our results show that *Bt407* induces a mild AMP-humoral response to which it is highly resistant. Surprisingly, this response is significantly enhanced and to the same level in flies infected by *Bt407*Δ*fla* or *Bt407*Δ*fliK* (Figure 3F). These results suggest that the absence of flagella makes *Bt407* more vulnerable to immune sensing in *Drosophila*. We do not presently have a clear understanding of how this is performed. However, as DAP-type PGN is known to be the major trigger of the immune response upon *Bacillus* spp. infection in *Drosophila* (Leulier et al., 2003), three scenarios have been proposed for their sensing by cognate host receptors: (1) accessibility of the receptor to cell wall PGN; (2) the release of PGN fragments through the activity of enzymes produced by bacteria, or by (3) host effector molecules such as AMPs or lysozyme (Chaput and Boneca, 2007; Humann and Lenz, 2009; Atilano et al., 2011; Vaz et al., 2019). We have so far verified whether PGN is already further released from the mutant *Bt407*Δ*fla* or *Bt407*Δ*fliK* bacteria as compared to the wt strain by dosing the IMD response triggered in flies injected with supernatant of bacterial overnight cultures. The results presented in Supplementary Figure 1A. A clearly indicate that the supernatant of both *Bt407*Δ*fliK* and *Bt407*Δ*fla* are more immunostimulatory than that of the wt or *Bt407*Δ*fliKΩfliK* complemented strains. This enhanced immunostimulatory effect is not due to an enhanced autolysis of the mutant strains (Supplementary Figure 1B). These results clearly suggest that the absence of flagella, somehow, perturbates the cell wall integrity thus resulting in an enhanced release of immunostimulatoty PGN fragments (Supplementary Figure 1A). The partial controlled growth of *Bt407*Δ*fla* by the AMP response it induces in the infected flies could be explained by the enhanced expression of these immune effectors by the host immune response. Nonetheless, we cannot exclude the possibility that a flagellar dependent mechanism would provide successfully growing bacteria an accentuated resistance to the accumulated production of AMPs *in vivo*.

Although our data put forward FliK as an essential element for the enhanced resistance of *B. thuringiensis* to AMPs, the molecular mechanism underlying this particular function remains to be clarified. The flagellar apparatus is a complex self-assembling nanomachine that contains its own type III protein export apparatus that has various substrate specificities. During flagellar morphogenesis, the export apparatus switches substrate specificity from the rod-/hook-type substrate to the filament-type substrate (Minamino et al., 1999a,b). In *B. subtilis*, this switch in export specificity also results in the export of the anti-sigma factor FlgM that inhibits late-class flagellar gene expression by sequestering a flagellar-specific sigma factor (Gillen and Hughes, 1991; Kutsukake and Iino, 1994; Daughdrill et al., 1997; Karlinsey et al., 2000a,b; Kalir et al., 2001; Barembruch and Hengge, 2007). Secreted FlgM is further degraded extracellularly by the proteases Epr and WprA (Calvo and Kearns, 2015). It is currently well known that FliK plays an essential role in switching the substrate specificity of the flagellar export apparatus by modifying the export gate proteins FlhA and FlhB that control flagellar protein export (Aizawa, 2012; Hughes, 2012a,b; Evans et al., 2014; Minamino, 2018; Minamino et al., 2019; Kinoshita et al., 2020). This modification allows specificity switch for the secretion among others of filament class proteins (Ghelardi et al., 2002; Bouillaut et al., 2005; Minamino, 2018). Based on these data, it is tempting to speculate that FliK might be involved in the expression or secretion of proteins/effectors that would account for the enhanced resistance of *B. thuringiensis* to AMPs. This hypothesis is supported by previous studies that have pinpointed the role of flagella in the secretion of virulence factors (Young et al., 1999; Duan et al., 2013). For example, in *B. thuringiensis* the expression of some secreted virulence determinants was shown to be dependent on a functional flagellar export apparatus (Bouillaut et al., 2005; Fagerlund et al., 2010). In particular, a *Bt407* mutant carrying a mutation of the *flhA* gene, which encodes an essential protein of the flagellar secretion apparatus, is defective for the secretion of some virulence factors and was shown to exhibit a decreased expression of these virulence factors and to be less virulent in the *Galleria mellonella* insect model by both force-feeding and intrahemocoelic injection (Ghelardi et al., 2002; Bouillaut et al., 2005; Fagerlund et al., 2010). Likewise, FlhA was proposed as defining element for the regulation of virulence factors in *C. jejuni* (Carrillo et al., 2004). We strongly believe that investigation of FliK’s cellular functions deserves a more detailed study and are aiming, in the future, to examine its impact on the molecular composition of the cell envelope and the regulation of the transcription/secretion program in *B. thuringiensis*. In sum, by identifying FliK as prerequisite for the resistance of *B. thuringiensis* to host AMPs, this study sets the way for in depth studies useful for the identification of potential novel targets for the development of new antibacterial therapeutic strategies.

## Materials and Methods

### Bacterial Strains, Growth Conditions, and Bacterial Products

The acrystalliferous strain *B. thuringiensis* 407 Cry-(*Bt407*) (Lereclus et al., 1989) was used as wild-type throughout this study. *E. coli* K-12 strain *TG1* was used as a host for cloning experiments. *E. coli* strain *ET12567 (dam^–^; dcm^–^)*, was used to generate unmethylated plasmid DNA for *Bt407* electrotransformation. *Bt407* and *E. coli* strains were transformed as previously described (Dower et al., 1988; Lereclus et al., 1989).

A *B. thuringiensis* strain containing a *fliK* deletion was generated by precise, in frame allelic exchange and deletion replacement without antibiotic resistance cassettes. The thermosensitive plasmid MAD (pMAD) was used in these experiments. The 1.253 kb sequence immediately upstream from *fliK* was amplified with the primers fliK-a and fliK-b, and a 1.217 kb sequence immediately downstream from *fliK* was amplified with the primers fliK-c and fliK-d. The primers fliK*-*b and fliK-c introduce overlapping PCR products. The two amplicons were then subjected to another PCR cycle with the primers fliK-a and fliK-d, such that an amplicon, from which the 1.096 kb of *fliK* gene has been deleted, was amplified. This amplicon was digested with BamHI and NcoI and was introduced between the corresponding cloning sites of pMAD. *Bt407* was then transformed with 10 μg of the recombinant plasmid by electroporation as previously described (Lereclus et al., 1989). Transformants were subjected to allelic exchange by homologous recombination and bacteria sensitive to erythromycin, resulting from double crossing over event in which the chromosomal *fliK* copy was replaced with the overlapping sequences, were selected. The procedure for selection of mutants by allelic exchange via double crossover has been described previously (Bravo et al., 1996). The chromosomal allelic exchange in the *fliK* mutant was checked by PCR using the appropriate primer couples (fliK-a and fliK-d) and confirmed by DNA sequencing of the PCR fragments generated from the primer pairs fliK-a and fliK-d. The resulting *fliK*-deficient strain was designated *Bt407*Δ*fliK*. For selection and CFU counting, we transformed *Bt407* and *Bt407*Δ*fliK* with the unmethylated plasmid pHT315paphA3gfp containing ampicillin and erythromycin resistance cassettes. The primers fliK-a, fliKb, fliKc, and fliK-d are listed in the Supplementary Table 1.

The complemented strain of *Bt407*Δ*fliK* was constructed as follows*:* From *Bt407* genomic DNA, the intergenic region immediately located upstream the operon, in which *fliK* is the second gene, was amplified and then cloned upstream the entire *fliK* gene by using the splicing by overlap extension technique (SOE). The primer CompfliK-a included a restriction site for BamHI and was used with CompfliK-b to amplify the intergenic region. The *fliK* gene was amplified from *Bt407* genomic DNA by using CompfliK-c as forward primer and CompfliK-d that included a restriction site for EcoRI as reverse primer. The primers CompfliK-b and CompfliK-c introduce overlapping PCR products, which then served as matrix to a final PCR using CompfliK-a and CompfliK-d as primers. The resulting 1.274 kb fragment consisting of the full-length *fliK* gene downstream the intergenic region was digested with BamHI and EcoRI, gel-purified, and ligated to the pHT304-18 shuttle vector previously digested with the same enzymes. An aliquot of ligation mixture (∼50 ng DNA) was used to transform *E. coli* K-12 strain *TG1* by electroporation as described previously (Lereclus et al., 1989). The resulting construct, pHT304-18Ω*fliK*, was verified by sequencing using the pHT304-18 specific primers PU and PR and then transferred into *E. coli ET12567* by electroporation. Unmethylated plasmid pHT304-18Ω*fliK* from *E. coli ET12567* was then introduced into strain *Bt407*Δ*fliK* by electroporation to generate the complemented *Bt407*Δ*fliKΩfliK* strain. Plasmid extraction was performed from the complemented strain, and the presence of pHT304-18Ω*fliK* was checked by sequencing using PU and PR primers. The primers CompfliK-a, CompfliK-b, CompfliK-c, CompfliK-d, PU, and PR are listed in Supplementary Table 1.

*Bt407*Δ*dltX* and *Bt407*Δ*dlt* have been described (Kamar et al., 2017; Attieh et al., 2019). *Bt407*Δ*fla* mutant was provided by Dr. Michel Gohar (Université Paris-Saclay, INRAE, AgroParisTech, Micalis Institute, Jouy-en-Josas, France) (Houry et al., 2010). *E. coli DH5αGFP* (Bonnay et al., 2013), commonly used as agent to induce *Drosophila* IMD pathway, was a kind gift from Pr. Jean Marc Reichhart (UPR9022—CNRS—Université de Strasbourg). All strains were grown in Luria-Bertani (LB) broth, with vigorous shaking, at 37°C for *E. coli* strains and at 30°C for *Bt407* strains. The antibiotic concentrations used for bacterial selection were as follows: 100 μg/ml of ampicillin for *E. coli*; 100 μg/ml of spectinomycin for *E. coli* and 300 μg/ml of spectinomycin for *B. thuringiensis* and 10 μg/ml of erythromycin for *Bt407* and its derivatives. β-Galactosidase production was detected on LB plates supplemented with X-Gal (5-bromo-4-chloro-3-indolyl-β-d-galactopyranoside) at 100 μg/ml.

Heat killing of bacteria was performed as described (El Chamy et al., 2008). Briefly, bacterial solutions followed two steps of 20 min of incubation at 95°C separated by 20 min of cooling on ice. Killing was verified by plating 100 μl of each bacterial solution on LB agar plates.

### DNA Manipulations

Chromosomal DNA was extracted from *B. thuringiensis* using the Puregene Yeast/Bact. Kit B (QIAgen, France). Plasmid DNA was extracted from *E. coli* by standard alkaline lysis by using QIAprep spin columns (QIAgen, France). Restriction enzymes and T4 DNA ligase were used as recommended by the manufacturer (New England Biolabs). Oligonucleotide primers (Supplementary Table 1) were synthesized by Sigma Proligo (Paris, France). PCRs were performed in an Applied Biosystems 2720 Thermal cycler (Applied Biosystem, United States) with Phusion High-Fidelity or Taq DNA Polymerase (New England Biolabs). Amplified DNA fragments were purified with the QIAquick PCR purification kit (QIAgen, France). Digested DNA fragments were separated by electrophoresis and purified from agarose gels using the QIAquick gel extraction kit (QIAgen, France). All constructions were confirmed by DNA sequencing by GATC Biotech (Konstanz, Deutschland).

### Generation of a *B. thuringiensis* Strain 407 (Cry^–^) Transposon Library

A mini-Tn*10* derivative of *Salmonella enterica serovar Typhimurium* Tn*10* transposon, carried out by the thermosensitive plasmid pIC333, as a transposon delivery system, was used for random insertion mutagenesis (Supplementary Figure 2; Petit et al., 1990; Steinmetz and Richter, 1994). Outside the transposon, the plasmid pIC333 contains the thermosensitive replication origin of pE194 (pE194 ts) plasmid, a gene conferring resistance to erythromycin (*erm*^*R*^) as well as a Tn*10* transposase allele *tnpA* with relaxed target specificity to increase the transposition randomness. The mini-Tn*10* transposon itself, in pIC333, is a 2.4 kb element composed of the two ends of Tn*10* flanking a spectinomycin resistance gene (*spec*^*R*^) and a pColE1-type origin of replication (Ori pColE1). An insertion library was constructed in *Bt407* as described previously (Gominet et al., 2001; Espinasse et al., 2002; Fedhila et al., 2004; Kamoun et al., 2009). After *Bt407* transformation with the pIC333 at 30°C, transformants were selected for their resistance to erythromycin. Subsequently, an upshift to the non-permissive temperature (37°C) under selection for spectinomycin resistance allowed the pIC333 elimination and the selection of derivatives in which the miniTn*10* had integrated into the chromosome. Briefly, the culture was diluted 1:100 into fresh LB medium only supplemented with spectinomycin (150 μg/ml), and grown overnight while shaking at 37°C. This step was repeated 6–8 times. Appropriate dilutions of the growing cells were then plated on LB agar containing Spectinomycin (150 μg/ml) and incubated at 40°C for the selection of insertion mutants. Approximately 20,000 spectinomycin-resistant colonies were randomly selected and stocked as individual clones at −80°C.

### Screening the *B. thuringiensis* Strain 407 (Cry^–^) Transposon Library for Sensitivity to Polymyxin B

The insertion mutants were then screened for increased sensitivity to polymyxin B, and those with higher sensitivity were selected. Appropriate dilutions of the mutant library were plated on LB agar medium supplemented with spectinomycin and incubated at 40°C overnight. All Spec^R^ clones were then replicated on LB—agar medium containing erythromycin. The Spec^R^ Erm^s^ clones (about 70% of the Spec^R^ clones were Erm^s^) were considered a mini-Tn*10* insertion mutant, which were selected, inoculated on LB-Agar Petri dishes supplemented or not with 200 μg/ml of polymyxin B (50 colonies per plate) and then incubated at 30°C for 48 h. *Bt407* and *Bt407*Δ*dltX* (Kamar et al., 2017) were used as positive and negative controls, respectively. Strains were considered as insensitive to 200 μg/ml of polymyxin B when colonies appeared after the above incubation times.

### Marker Rescue of Flanking Genomic DNA and Identification of Transposon Insertion Site

In order to determine the mini-Tn*10* insertion site within *Bt407* mutant clones’ genome, a plasmid rescue has been conducted. Chromosomal DNA was extracted, digested with *Eco*RI or HindIII (The mini-Tn*10* element contains no restriction sites for these enzymes) and the digestion products were self-ligated using T4 DNA ligase. *E*. *coli TG1* cells were than transformed with the ligation mixture, hence allowing the selection of transformed clones with a plasmid containing the mini-Tn*10* element and the DNA fragments flanking the insertion locus (The mini-Tn*10* element harbors an *E*. *coli* replicon and a gene conferring resistance to spectinomycin). Putative clones obtained by selection for their resistance to spectinomycin were subjected to plasmid DNA extraction. In order to confirm the presence of mini-Tn*10* in the selected clones genome, the restriction map of the plasmid was determined by the presence of a 2.2 kb BamHI characteristic fragment of mini-Tn*10.* As to determine the mini-Tn*10* insertion site, the chromosomal DNA flanking the insertion locus in the plasmid was sequenced, using primers E1 and E3 (Supplementary Table 1) matching the transposon extremities. Sequencing reads were mapped to *Bt407* genome by using the BLAST program of the National Center for Biotechnology Information Genbank (NCBI).

### Atomic Force Microscopy (AFM) Analysis

Bacteria were visualized by AFM. The strains *Bt407*, *Bt407*Δ*fliK*, and *Bt407*Δ*fliKΩfliK* were taken from a frozen stock and grown overnight in 5 ml of liquid LB medium at 30°C and 220 rpm. The next day, cultures were diluted to 10^–6^ and 100 μl of each was spread on LB plates and incubated overnight at 30°C. One colony of each of the strains was then inoculated in 5 ml of liquid LB medium and grown overnight at 30°C and 220 rpm. The next day, 0.2 ml of each of the three strain cultures was inoculated into 20 ml of LB and grown as described above; samples were withdrawn for density measurements at 30 min intervals, until an OD_600_ of 1.0 was reached. One millilitre from each culture was then centrifuged at 4,000 rpm for 3 min. The supernatant was thrown away, and the pellet carefully resuspended in 1 ml PBS. Bacterial cells were centrifuged once more and resuspended again, in 1 ml MilliQ water. Twenty microlitre of sample was then added to 5 μl (100 mM) Tris/Mg^2+^ buffer and 25 μl MilliQ water. Ten microlitre of this solution was applied on a newly cleaved Mica (glued to a microscope slide), incubated at room temperature for 10 min, and then washed 10 times with 100 μl MilliQ water, before being air dried for some minutes and finally dried with a soft N_2_ gas stream. The slide was then scanned in an Atomic force microscope (JPK NanoWizard instrument, Berlin, Germany). The preparations were scanned using Intermittent contact (Ic) mode and a Silicon NSC35/AlBS cantilever (MicroMasch Spain). All scans were performed in air. The Error images obtained in Ic mode are named Amplitude images.

### Sensitivity to Antimicrobial Peptides

Polymyxin B was used as a prototype antimicrobial compound for the large-scale screening of the collection. The test was performed in 96-well microplates containing seven concentrations of polymyxin B (Sigma) from 200 to 800 μg/ml for *Bt407, Bt407Δfla*, and *Bt407*Δ*fliKΩfliK* and from 25 to 250 μg/ml for *Bt407*Δ*fliK*. Bacterial growth was scored after inoculation with strains at an initial OD_600_ = 0.1 and incubation at 30°C for 6 h. Susceptibility to polymyxin B was evaluated by determining the half inhibitory concentration (IC_50_), corresponding to the concentration of polymyxin B halving inoculum viability. IC_50_ was determined by examining the dose-response curves obtained with the various concentrations of polymyxin B.

### Motility Assay

The swimming ability of *Bt407* strains was determined on LB soft agar plates (0.25% agar, final concentration) by measuring the bacterial growth area. A volume of 5 μl of an OD_600_ = 1 bacterial culture was spotted in the agar plate center and then incubated at 37°C. The colony diameters were measured every 6 h.

### Biofilm Formation

The ability of *Bt407* and its derivatives to form biofilms was determined as previously described (Fagerlund et al., 2014). Briefly, cultures in the exponential phase were diluted into HCT medium to an OD_600_ of 0.01. UV-sterilized 6 ml glass tubes were inoculated with 2 ml of the diluted cultures and then incubated for 48 h at 30°C without shaking. At the end of the incubation time, the 2 ml culture medium was removed using a Pasteur pipette and the OD_600_ of the biofilm, thoroughly vortexed in 2 ml PBS, was measured.

### Autolysis Test

The autolysis test was performed as previously described (Abi Khattar et al., 2009). Briefly, bacterial solutions prepared from bacterial cells in exponential growth phase, washed twice with cold PBS, and resuspended in the same buffer supplemented with 0.05% Triton X-100, were incubated in 96-well plate at 37°C without shaking and autolysis was monitored by measuring the OD_600_ every 30 min.

### *Drosophila melanogaster* Stocks and Maintenance

DrosDel isogenic w^1118^ (*iso w^1118^*) (Ryder et al., 2004) and Oregon^R^ were used as wild-type. The antimicrobial peptides mutant (Δ*AMPs*) and its wt control *iso w^1118^* (Hanson et al., 2019) are a kind gift from Pr. Bruno Lemaitre (Global Health Institute, School of Life Sciences, École Polytechnique Fédérale de Lausanne, Lausanne, Switzerland). The IMD pathway mutant *relish*^*E20*^ (*Rel*) has been described (Hedengren et al., 1999). Fly stocks were raised on cornmeal-agar medium rich in yeast (7.25%) at 25°C.

### Survival Experiments, Injections, and Immune Induction

Survival experiment were performed on a total of ≥ 45 adult females per genotype (15–20 individuals per each of the three biological replicates). Batches of 15–20 female flies, aged between 2 and 4 days old, were pricked with a tungsten needle previously dipped into a bacterial solution prepared from an overnight culture that was washed and diluted in PBS (1×) to a final OD_600_ = 2. The infected flies were incubated at 29°C. The *Diptericin* expression was measured by RT-qPCR 4 h post infection.

Sixty nine nanoliter of culture supernatant were injected into the thorax of batches of 20–25 female flies (aged 2–4 days old) with a Nanoject apparatus (Drummond, Broomall, PA). These flies were incubated 4 h at 29°C then *Diptericin* expression was quantified in RNA extracts by RT-qPCR.

### Fly Internal Bacterial Load Quantification

The CFU counting was performed by plating serial dilutions of lysates obtained from 10 infected flies as described previously on LB agar medium containing the appropriate antibiotic to each strain.

### Quantitative Real Time PCR

Total RNA was extracted with TRI Reagent (Sigma-Aldrich) from 15 to 25 adult of each genotype per biological replicate. The reverse transcription was performed on 1 μg of RNA by using the RevertAid RT Reverse Transcription Kit (Thermo Fisher Scientific). cDNA was used as template in PCR reaction with three 10 μl technical replicates of each biological sample. qPCR was performed on an iQ5 Real Time PCR detection system (Biorad) using iTaq Universal Syber Green supermix (Biorad). The amount of RNA detected was normalized to that of the house keeping gene *rp49*. Primers for *Diptericin* and *rp49* genes are listed in the Supplementary Table 1. Relative gene expression levels between control and experimental samples was determined using the ΔΔCT method. Each experimental sample was compared to each wt sample.

### Statistical Analysis

All data analysis was performed using GraphPad Prism 8.0.2 software and statistical tests used for each data set are indicated in figure legends.

## Data Availability Statement

The original contributions presented in the study are included in the article/Supplementary Material, further inquiries can be directed to the corresponding author/s.

## Author Contributions

ZA conceived, designed and performed the experiments, analyzed the data, contributed to the manuscript writing, and reviewed and edited the manuscript. CM, AR, and IJ performed the experiments. SP designed and constructed the *Bt407*Δ*fliK* mutation. IH and OØ conceived experiments, analyzed data, and reviewed and edited the manuscript. MKA conceived and designed the experiments, supervised the work, analyzed the data, and reviewed and edited the manuscript. VS-B and LEC conceived and designed the study, conceived and designed the experiments, supervised the work, analyzed the data, and wrote the manuscript. All authors contributed to the article and approved the submitted version.

## Conflict of Interest

The authors declare that the research was conducted in the absence of any commercial or financial relationships that could be construed as a potential conflict of interest.
