# Supplementary material for: The fliK Gene Is Required for the Resistance of Bacillus thuringiensis to Antimicrobial Peptides and Virulence in Drosophila melanogaster
Source: Front Microbiol. 2020 Dec 18;11:611220. doi: 10.3389/fmicb.2020.611220 (PMC7775485; doi:10.3389/fmicb.2020.611220)
Supplement: Supplementary file 1 [file Data_Sheet_1.pdf]

frontiers

Supplementary Material

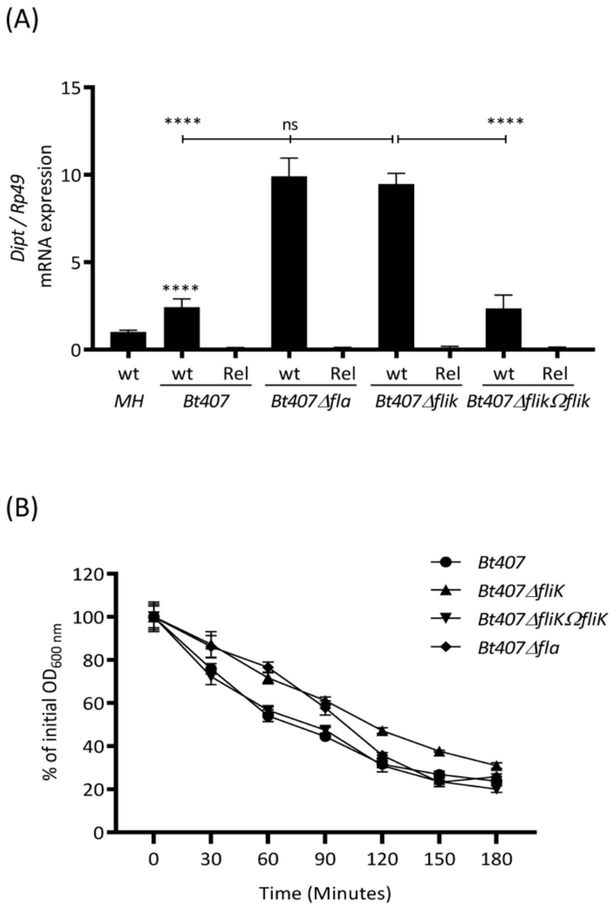

Supplementary Figure 1. The loss of *Bacillus thuringiensis* flagellar filaments enhances the release of immunostimulatory fragments of peptidoglycan. (A) Wild-type (wt) and *relish* (Rel) mutant flies were injected with the supernatant of *Bt407, Bt407ΔfliK, Bt407ΔfliKΩfliK or Bt407Δfla* cultures. Relative *Diptericin (Dipt*) expression was measured by RT-qPCR 4 h upon the induction. Ribosomal protein 49 (Rp49) transcript was used as reference gene. Transcripts levels are compared to that triggered in wt flies injected with Muller Hinton broth (MH) as a control. **(B)** Autolysis rates of *Bt407*, *Bt407ΔfliK*, *Bt407ΔfliKΩfliK* and *Bt407Δfla* in the presence of Triton X-100. Autolysis rates were determined over a period of 3 h (180 min) in PBS at 37 °C, without shaking. Data obtained from three independent experiments are combined in single value (mean ± sd). Statistical tests were performed using the Mann-Whitney test within Prism software (ns: $p > 0.05$; ****: $p < 0.0001$).

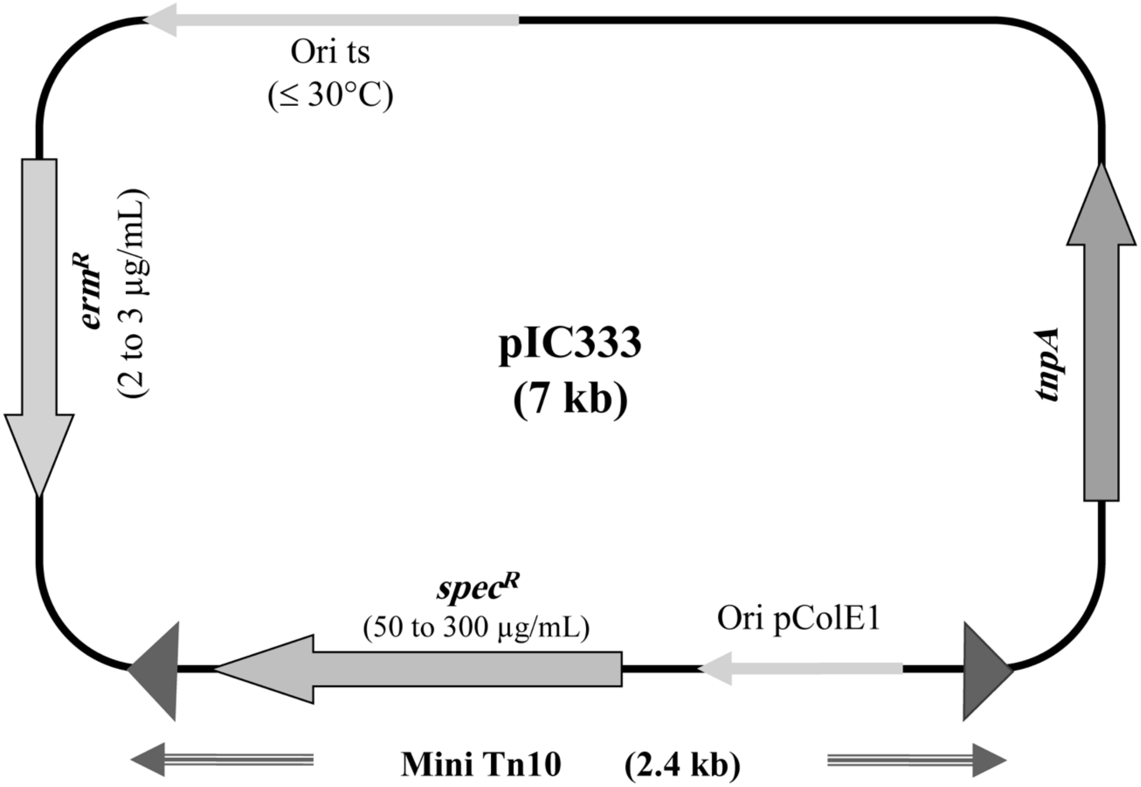

Supplementary Figure 2. Map of the temperature sensitive plasmid pIC333. The plasmid pIC333 carries the Gram-positive thermosensitive replication origin of pE194 plasmid (Ori ts), a gene conferring resistance to erythromycin (*erm*^{R}), a Tn*10* transposase gene (*tnpA*) and the transposable element mini-Tn*10*. The mini-Tn*10* (double grey arrow) is a 2.4 kb transposon flanked by inverted terminal repeat ends (dark grey triangles), containing a pColE1-type origin of replication (Ori pColE1) and a gene coding for resistance to spectinomycin (*spec*^{R}). This transposon system was originally used to generate random *B. subtilis* mutants (Steinmetz & Richter, 1994).

Supplementary Table 2. Primers sequences used in this study.

Name	Nucleotide sequence (5'→3')	Restriction site
fliK-a	CGCGGATCCGAACGTGGTCGGGAAGTAAA	BamHI
fliK-b	CCAACTGTTGGCACGCTTTATTCCTCCAATATCATCCATTCATACACATTTTCAAATCCAG	None
fliK-c	CTGGATTTGAAAATGTGTATGAATGGATGATATTGGAGGAATAAAGCGTGCCAACAGTTGG	None
fliK-d	CATGCCATGGCGCATTGTCCATGTATTCCC	NcoI
CompfliK-a	CGCGGATCCGATGATATCGTTGAAGCGATACACC	BamHI
CompfliK-b	GGTAATACAGACTGTATCACCTGTTTTCATAGCCCCTTCC	None
CompfliK-c	GGAAGGGGCTATGAAAACAGGTGATACAGTCTGTATTACC	None
CompfliK-d	CCGGAATTCTTATTCCTCCAATAATCCCGC	EcoRI
E1	CGTTGGCCGATTCATTAATGC	None
E3	CGATATTCACGGTTTACCCAC	None
PU	CGCCAGGGTTTTCCCAGTCACGAC	None
PR	AGCGGATAACAATTTCACACAGGA	None
DiptRTFw	GCTGCGCAATCGCTTCTACT	None
DiptRTRv	TGGTGGAGTGGGCTTCATG	None
Rp49RTFw	GACGCTTCAAGGGACAGTATCTG	None
Rp49RTRv	AAACGCGGTTCTGCATGAG	None
